# *Myo*‐inositol‐1‐phosphate synthase (Ino‐1) functions as a protection mechanism in *Corynebacterium glutamicum* under oxidative stress

**DOI:** 10.1002/mbo3.721

**Published:** 2018-10-01

**Authors:** Can Chen, Keqi Chen, Tao Su, Bing Zhang, Guizhi Li, Junfeng Pan, Meiru Si

**Affiliations:** ^1^ College of Life Sciences Qufu Normal University Qufu Shandong China; ^2^ Institute of Food and Drug Inspection College of Life Science and Agronomy Zhoukou Normal University Zhoukou Henan China; ^3^ Shaanxi Key Laboratory of Agricultural and Environmental Microbiology College of Life Sciences Northwest A&F University Yangling Shaanxi China

**Keywords:** *Corynebacterium glutamicum*, *Myo*‐inositol‐1‐phosphate synthase (Ino‐1), oxidative stress, protein carbonylation

## Abstract

Reactive oxygen species (ROS) generated in aerobic metabolism and oxidative stress lead to macromolecules damage, such as to proteins, lipids, and DNA, which can be eliminated by the redox buffer mycothiol (AcCys‐GlcN‐Ins, MSH). *Myo*‐inositol‐phosphate synthase (Ino‐1) catalyzes the first committed step in the synthesis of MSH, thus playing a critical role in the growth of the organism. Although Ino‐1s have been systematically studied in eukaryotes, their physiological and biochemical functions remain largely unknown in bacteria. In this study, we report that Ino‐1 plays an important role in oxidative stress resistance in the gram‐positive *Actinobacteria Corynebacterium glutamicum*. Deletion of the *ino‐1* gene resulted in a decrease in cell viability, an increase in ROS production, and the aggravation of protein carbonylation levels under various stress conditions. The physiological roles of Ino‐1 in the resistance to oxidative stresses were corroborated by the absence of MSH in the Δ*ino‐1* mutant. In addition, we found that the homologous expression of Ino‐1 in *C. glutamicum* yielded a functionally active protein, while when expressed in *Escherichia coli* BL21(DE3), it lacked measurable activity. An examination of the molecular mass (Mr) suggested that Ino‐1 expressed in *E. coli* BL21(DE3) was not folded in a catalytically competent conformation. Together, the results unequivocally showed that Ino‐1 was important for the mediation of oxidative resistance by *C. glutamicum*.

## 1 INTRODUCTION

As a consequence of living in an aerobic world, organisms inevitably meet the challenge of oxidative stress and maintaining a good cellular redox state. Oxidative stress is generated by imbalances between the production and clearance of reactive oxygen species (ROS), damaging nucleic acids, proteins, carbohydrates, and lipids (Carmel‐Harel & Storz, 2000; González‐Flecha & Demple, 2000; Wheeler & Grant, 2004). Thus, protection against oxidative stress is likely to play an important role in facing the challenges of changing environments and surviving under conditions of stress.

Microbes have evolved complex systems for sensing, protecting against, and regulating toxicity, as well as minimizing the adverse effects of and repairing the damage caused by ROS (Anderson, 1998; Dalle‐Donne et al., 2008; Kim, Shin, Kim, Kim, & Yoon, 2009; Newton et al., 1995). One of these systems is to synthesize low‐molecular‐weight (LMW) thiols that act as redox buffers in the defense against ROS and modify proteins with the help of their own cysteines to maintain the reduced state of the cytoplasm and protect important proteins (Antelmann & Helmann, 2011; Van Laer, Hamilton, & Messens, 2013). Eukaryotes and gram‐negative bacteria produce the tri‐peptide glutathione (γ‐l‐glutamyl‐l‐cysteinylglycine, GSH) as the LMW thiol redox buffer, while some gram‐positive bacteria, such as members of *Corynebacterium*,* Mycobacterium*,* Rhodococcus,* and *Streptomyces*, cannot produce GSH but instead synthesize its functional equivalent, mycothiol (MSH, AcCys‐GlcN‐Ins) (Anderson, 1998; Newton, Buchmeier, & Fahey, 2008). Like GSH, MSH plays a key role in protecting the cell against environmental stresses, such as antibiotics, alkylating agents, oxidants, heavy metals, and extreme pH (Eggeling & Sahm, 1985; Liu et al., 2013; Newton, Fahey, & Rawat, 2012; Newton et al., 1996). Therefore, MSH is considered to be a general protective agent to improve the survival of cells facing environmental stress conditions in some kinds of bacteria, including *C. glutamicum*.

When subjected to environmental stress, MSH is consumed to confront the adverse effects. To generate more MSH in *Corynebacterineae* (including *C. glutamicum* and *Mycobacterium tuberculosis*), *myo*‐inositol‐1‐phosphate synthase (Ino‐1) has been evolutionarily used to synthesize the key precursor substrate (*myo*‐inositol‐phosphate, Ins‐P) of MSH but is independent from phosphatidylinositol, glycosylphosphatidylinositol anchors linked to complex carbohydrates, the cell membrane and lipids (Bachhawat & Mande, 1999; Krings et al., 2006; Newton, Ta, Bzymek, & Fahey, 2006; Newton et al., 2003), implicating the possible role of Ino‐1 in stress resistance. In this study, we systematically examined the physiological roles of Ino‐1 in response to oxidative stress by removing the *ino‐1* gene in *C. glutamicum*. We present the evidence that *C. glutamicum* Ino‐1 protects against the damaging effects of ROS induced by various exogenous oxidative stresses via modulating the levels of MSH.

## 2 MATERIALS AND METHODS

### 2.1 Bacterial strains and culture conditions

The bacterial strains and plasmids used in this study are listed in Supporting Information Table S1. *C. glutamicum* and *Escherichia coli* strains were cultured in Luria‐Bertani (LB) broth aerobically on a rotary shaker (220 rpm) or on LB plates at 30 or 37°C, respectively, as previously reported (Shen, Jiang, Huang, Liu, & Liu, 2005). Mineral salts' medium (Shen et al., 2005) containing 100 mM glucose or 50 mM *myo‐*inositol (*mIno*) as the sole carbon source was used to determine the content of the MSH and Ins‐P and bacterial growth with or without H_2_O_2_ as previously described. To construct the Δ*ino‐1* in‐frame deletion mutants, the pK18*mobsacB*‐Δ*ino‐1* plasmids were transferred into *C. glutamicum* by electroporation, and chromosomal integration was selected by single crossover on LB agar plates containing 25 μg/ml kanamycin and 40 μg/ml nalidixic acid. After the kanamycin‐resistant (Km^R^) colonies (the resulting chromosomal integration strains) were cultured overnight in LB for a second crossover, the Δ*ino‐1* deletion mutants were subsequently screened on LB agar plates containing 20% sucrose and 40 μg/ml nalidixic acid and confirmed by PCR and DNA sequencing as previously described (Shen et al., 2005). For complementation in relevant *C. glutamicum* strains, the pXMJ19 or pXMJ19‐His_6_ derivatives were transferred into relevant *C. glutamicum* strains by electroporation, and the expression in *C. glutamicum* was induced by the addition of 0.5 mM isopropyl β‐d‐1‐thiogalactopyranoside (IPTG). All enzymes were purchased from Sigma‐Aldrich (St. Louis, MO). All antibiotics were purchased from Gold Biotechnology (Shanghai, China). All chemicals, oxidants, and heavy metals were purchased from Aladdin (Shanghai, China). When needed, antibiotics were used at the following concentrations: kanamycin, 50 μg/ml for *E. coli* and 25 μg/ml for *C. glutamicum*; nalidixic acid, 40 μg/ml for *C. glutamicum*; chloramphenicol, 20 μg/ml for *E. coli* and 10 μg/ml for *C. glutamicum*.

### 2.2 Plasmids construction

To obtain heterologous expression plasmids, the gene encoding *ino‐1* (*cg3323*) was amplified by PCR using *C. glutamicum* genomic DNA as a template with Ino‐1‐F/Ino‐1‐R primers, as listed in Supporting Information Table S1. The amplified DNA fragments were digested and cloned into a similarly digested pET28a plasmid, obtaining plasmid pET28a‐*ino‐1*. To construct the deletion plasmid pK18*mobsacB*‐Δ*ino‐1*, the primer pairs DIno‐1‐F1/DIno‐1‐R1 and DIno‐1‐F2/DIno‐1*‐*R2 were used to obtain a 1,006‐bp upstream fragment and a 997‐bp downstream fragment, respectively (Supporting Information Table S1). The upstream and downstream PCR fragments were fused together with the primer pair DIno‐1‐F1/DIno‐1*‐*R2 by overlap extension PCR (Si, Xu, et al., 2015). The resulting DNA fragments were digested with *Xbal*I/*EcoR*I and inserted into the similarly digested suicide plasmid pK18*mobsacB* to create pK18*mobsacB*‐Δ*ino‐1*. To obtain the homologous expression plasmid, the gene *ino‐1* was amplified using the primers Ino‐1‐F/Ino‐1‐R, digested, and cloned into similarly digested pXMJ19 and pXMJ19‐His_6_ vectors to produce pXMJ19‐*ino‐1* and pXMJ19‐His_6_‐*ino‐1* plasmids.

### 2.3 Sensitivity assays for oxidants, alkylating agents, and heavy metals

Sensitivity assays for various stress conditions were investigated as previously described (Si, Xu, et al., 2015; Si, Zhang, et al., 2015). Overnight‐grown cultures of *C. glutamicum* (LB broth, 30°C) were diluted 100‐fold with LB medium and exposed to CHP (11 mM), MD (4 mM), H_2_O_2_ (100 mM), CdCl_2_ (300 μM), NiSO4 (6 mM), IAM (40 mM), MG (10 mM), and CDNB (70 mM) at 30°C with shaking for 30 min. After treatment, the cultures were serially diluted, spread on LB plates, and incubated at 30°C for 36 hr. Percentage survival was calculated as [(CFU/ml with stress)/(CFU/ml without stress)] × 100.

### 2.4 Susceptibility assay for antibiotics

Growth curves were measured by counting CFU/ml as previously described (Liu et al., 2014). Overnight cultures of *C. glutamicum* strains (OD_600_≈1.6) were centrifuged at 10,000 × *g* for 1 min. Cell pellets were resuspended in 1 ml LB medium and stressed with different concentrations of various antibiotics. After the cultures were treated at 30°C with agitation at 100* *rpm for 1 hr, the colony‐forming units were determined by using serial dilutions in triplicate on LB agar plates after treatment. After 36 hr, the survival rates were calculated by dividing the number of CFU of the stressed cells by the CFU of the unstressed control cells.

### 2.5 Bacteria growth under H_2_O_2_ stress

Bacterial growth under H_2_O_2_ stress was measured as previously described (Chi et al., 2013; Movahedzadeh et al., 2004; Shondorp & Matthews, 2004). Briefly, cells of the strain growing in the stationary phase in glucose‐minimal medium were diluted into the same medium at a 1:100 ratio. Cultures were allowed to grow to an OD_600_ of approximately 0.4 and treated with or without H_2_O_2_. After 30 min of H_2_O_2_ stress exposure, 100 μM MSH, *mIno*, or Ins‐P was added to the cultures, and the cellular growth was monitored by determining optical density at 600 nm.

### 2.6 Overexpression and purification of recombinant Ino‐1

Heterologous expression and the purification of the His_6_‐tagged proteins were performed as previously described (Si, Xu, et al., 2015; Si et al., 2016). Recombinant pET28a derivatives were transformed into *E. coli* BL21(DE3) host strains. The bacteria were grown at 37°C in LB medium to an OD_600_ of 0.5, shifted to 22°C and then induced with 0.5 mM IPTG, and cultivated for an additional 12 hr at 22°C. Harvested cells were disrupted by sonication and purified with Ni‐NTA His·Bind resin (Novagen, Madison, WI, USA) according to the manufacturer's instructions. The protein concentration was measured using the Bradford assay (Bio‐Rad, Hercules, CA) according to the manufacturer's instructions with bovine serum albumin (BSA) as the standard. For homologous expression, the pXMJ19‐His_6_ derivatives were transformed into Δ*ino‐1* mutants, and recombinant wild‐type His_6_‐Ino‐1 proteins were purified using the His·Bind Ni‐NTA resin (Novagen, Madison, USA) as described previously (Si, Xu, et al., 2015; Si et al., 2016).

### 2.7 Enzyme assay

Activity assays were performed based on the methods of the periodate assay as previously described with slight modification (Donahue et al., 2010; Huang & Hernick, 2015). Briefly, the assay uses NaIO_4_ to oxidize and chemically releases phosphate from the Ins‐P product. Assay mixtures (100 mM Tris–acetate, 2 mM DTT pH 7.5, 20 mM NH_4_‐Cl, 1 μM Ino‐1, 500 μM NAD^+^, and 50 μM ZnSO_4_) were preincubated at 30°C for 5 min, and the reactions were initiated with the addition of Glc‐6‐P (0‐100 mM). After 1 hr, the reaction mixtures were quenched by the addition of 20% trichloroacetic acid, and the phosphate group of Ins‐P was released by the addition of 0.2 M NaIO_4_. The released phosphate group was detected following reaction with ammonium molybdate/ascorbic acid by monitoring the increase in absorbance at 820 nm. The rate of phosphate production (μM/min) was calculated from a phosphate standard curve. The enzyme activity was determined after subtracting the rate of phosphate production detected with BSA from the micromolar amount of phosphate production (μM) per minute per μM Ino‐1 (i.e., turnover number, min^−1^). As a negative control, BSA (1 μM) was used in place of Ino‐1 in the same conditions. Three independent experiments were performed at each substrate concentration. The *k*_cat_ and *K*_m_ values of Ino‐1 were acquired from a nonlinear fit with the Michaelis‐Menten equation using the program GraphPad Prism 5.

The affinity of NAD^+^ for Ino‐1 was measured by monitoring the decrease in intrinsic Ino‐1 fluorescence intensity upon NAD^+^ binding (Chen, Zhou, Yang, & Roberts, 2000; Neelon, Wang, Stec, & Roberts, 2005). In brief, NAD^+^ (0–5 mM) was added to a solution of Ino‐1 (50 μM) in buffer (100 mM HEPES, 400 μM EDTA, pH 7.5). The fluorescence signal of the enzyme mixture (Excitation wavelength = 280 nm, Emission wavelength = 334 nm) was recorded following incubation at room temperature for 5 min in the dark. The decrease in the intrinsic fluorescence of Ino‐1 upon the addition of NAD^+^ was plotted as Δ*Ifl* = *I*_0_ − *I* (Δ*Ifl*, decrease in Ino‐1 intrinsic fluorescence; *I*_0_, fluorescence of Ino‐1 before the addition of NAD^+^; *I*, fluorescence of Ino‐1 after the addition of NAD^+^). The apparent *K*_*D*_ of Ino‐1 for NAD^+^ was obtained by fitting the following equation:

$$\Delta \mathrm{Fl}=\Delta {\mathrm{Fl}}_{\max}\times \frac{{[{\text{NAD}}^{+}]}_{\mathrm{Total}}}{(K_{D}+{[{\text{NAD}}^{+}]}_{\mathrm{Total}})}+\Delta {\mathrm{Fl}}_{\mathrm{initial}}$$

### 2.8 Examination of redox and oligomerization states

The oligomerization state of Ino‐1 was analyzed using the method previously described (An et al., 2011). The mixtures of the purified Ino‐1 and the loading buffer [250 mM Tris‐HCl (pH 6.8), 0.5% bromophenol blue (BPB), and 50% (v/v) glycerol] were separated on 12% PAGE and stained with Coomassie Brilliant Blue or detected by western blotting with anti‐his antibody.

The oligomerization state of the Ino‐1 was also determined using the gel filtration method on a Superdex 200 10/300 GL column connected to an FPLC system (GE Healthcare, Piscataway, NJ) as described previously (Si, Xu, et al., 2015). Fifty microliters of proteins (3.0 mg/ml) was loaded onto the column pre‐equilibrated in 50 mM potassium phosphate buffer (pH 7.2) containing 0.15 M NaCl. The flow rate stetted as 0.25 ml/min, and the absorbance was monitored at 280 nm. The molecular mass standards used were thyroglobulin (bovine) (670 kDa), γ‐globulin (bovine) (158 kDa), ovalbumin (chicken) (44 kDa), myoglobin (horse) (17 kDa), and vitamin B_12_ (1.35 kDa) (GE Healthcare, Piscataway, NJ). The *K*_av_ values for each of the standard protein were calculated using the equation *K*_av_ = (*V*_e_–*V*_0_)/(*V*_c_–*V*_0_), where *V*_0_ = column void volume, *V*_e_ = elution volume, and *V*_c_ = geometric column volume (24 ml). The elution volume of 8.72 ml for Blue Dextran 2000 is equal to the column void volume (*V*_0_). The plots of the *K*_av_ values obtained from the known standard protein were plotted against the log of the molecular mass of the standard protein to form the calibration curve. These data were suited with a linear equation. The molecular weight of an unknown protein could be determined from the calibration curve once its *K*_av_ value was calculated from its elution volume.

### 2.9 Measurement of intracellular ROS levels

To quantify the ROS levels in vivo, the 2′,7′‐dichlorofluorescein diacetate (DCFH‐DA)‐based assay was used as previously described (Si et al., 2016). The protein carbonylation assays were performed as previously described (Vinckx et al., 2011).

### 2.10 MSH purification and content determination

MSH was purified from *C. glutamicum* RES167 using thiopropyl sepharose 6B followed by Sephadex LH‐20 chromatography as previously described (Yin et al., 2010). The MSH concentration was determined using the thiol‐specific fluorescent‐labeling high‐performance liquid chromatography (HPLC) method as previously described (Feng et al., 2006; Yin et al., 2010). The HPLC used in this study was equipped with an Extend‐C18 column (ZORBAX, 250 × 4.6 mm) and was operated with aqueous acetic acid‐methanol gradient elution (eluent flow rate of 0.9 ml/min). The bimane derivative of MSH was eluted at approximately 15 min in this system.

### 2.11 In vivo *myo*‐Inositol‐phosphate content determination

Cells were grown overnight to an OD_600_ of 1.6 in glucose‐minimal medium at 30°C in a shaking incubator. The culture was divided into two parts: One was exposed to oxidative agents for 30 min at 30°C at 100* *rpm, while the other was grown without treatment (negative control). After treatment, the cells were harvested and resuspended in PBS buffer prior to sonication on ice. The cellular extracts were obtained by centrifugation. The resulting supernatants, diluted when necessary, were assayed for inositol phosphate content by the released phosphate group with the procedure described above (Barnett, Brice, & Corina, 1970; Donahue et al., 2010; Huang & Hernick, 2015).

### 2.12 Western blot analysis

Western blot analysis was performed as previously described (Si et al., 2016). Samples subjected to SDS‐PAGE were transferred onto polyvinylidene fluoride (PVDF) membranes. After blocking with 4% (w/v^−1^) milk for 2 hr at room temperature, membranes were incubated with the his primary antibody at 4°C overnight (Zhongshan Gold Bridge, Beijing). The blots were washed with 0.2% (v/v^−1^) Tween 20‐containing PBST buffer and incubated with horseradish peroxidase‐conjugated secondary antibody (Shanghai Genomics Inc., Shanghai, China). The protein bands were visualized with an ECL plus kit (GE Healthcare, Piscataway, NJ).

### 2.13 Statistical analysis

Statistical analyses of the survival rate, ROS level, and enzyme activity were determined using a paired two‐tailed Student's *t* test. GraphPad Prism Software was used to carry out the statistical analyses (GraphPad Software, San Diego, California, USA).

## 3 RESULTS

### 3.1 The *ino‐1* deletion mutant shows a sensitive phenotype under oxidants, alkylating agents, heavy metals, and antibiotic stress conditions

**Figure 1:**
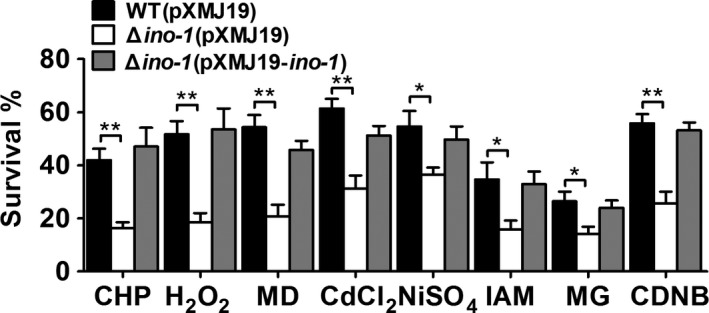
Ino‐1 was required for cellular resistance to oxidants, heavy metals, and alkylating agent‐induced stresses in *C. glutamicum*. The *C. glutamicum* WT (pXMJ19), Δ*ino‐1* pXMJ19), and Δ*ino‐1*(pXMJ19‐*ino‐1*) strains were exposed to various oxidants, including CHP (11 mM), H_2_O_2_ (100 mM), MD (4 mM), different heavy metals including CdCl_2_ (300 μM) and NiSO_4_ (600 μM), and different alkylating agents containing IAM (40 mM), MG (10 mM), and CDNB (70 mM) at 30°C for 30 min. Mean values with standard deviations (error bars) from at least three independent experiments are shown. n.s.: not significant. *p ≤ 0.05. **p ≤ 0.01

To address the question of whether Ino‐1 can protect *C. glutamicum* cells against diverse stresses, the wild‐type (WT) *C*. *glutamicum*, Δ*ino‐1* mutant, and complementary strains were challenged in the presence of various agents. As shown in Figure 1 and Supporting Information Figure S1, the growth of the Δ*ino‐1* mutant was nearly identical to the wild‐type and complementary strains under normal conditions, while the *ino‐1* deletion mutant was more sensitive to ROS‐inducing agents [cumene hydroperoxide (CHP), hydrogen peroxide (H_2_O_2_), menadione (MD), cadmium chloride (CdCl_2_), nickel sulfate (NiSO_4_), iodoacetamide (IAM), methylglyoxal (MG), and 1‐chloro‐2,4‐dinitrobenzene (CDNB)] than the WT (pXMJ19), and the survival rates of the Δ*ino‐1* mutant decreased by approximately 47%–70% compared to that of the wild‐type cells (Figure 1), similar to the results that Tan, Wang, Xiang, Han, and Guo (2013) reported for Ino‐1. Notably, the sensitivity phenotype of the mutants was almost completely rescued in the complementary strains Δ*ino‐1*(pXMJ19‐*ino1*) (Figure 1).

**Figure 2:**
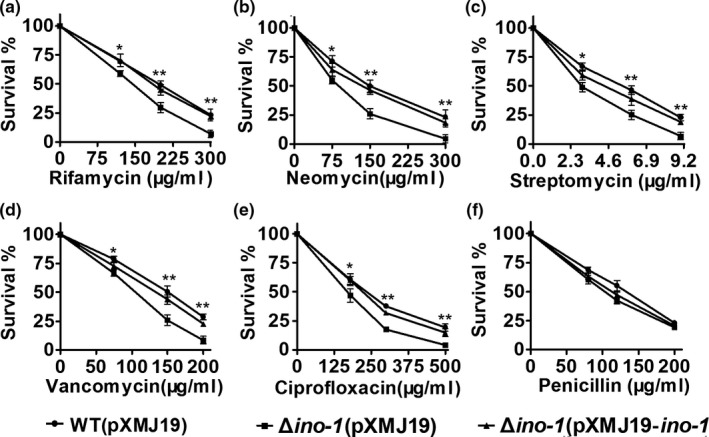
Survival of the *C. glutamicum* WT(pXMJ19), Δ*ino‐1*(pXMJ19), and Δ*ino‐1*(pXMJ19‐*ino‐1*) strains after challenge with different antibiotics. Survival of the *C. glutamicum* WT (pXMJ19), Δ*ino‐1* (pXMJ19), and Δ*ino‐1* (pXMJ19‐*ino‐1*) strains after challenge with different concentrations of rifamycin SV, ciprofloxacin, streptomycin, vancomycin, neomycin, and penicillin for 1 hr. Mean values with standard deviations (error bars) from at least three independent experiments are shown. *p ≤* *0.05. **p ≤* *0.01

It has been proposed that antibiotics can contribute to oxidative cellular conditions through a common mechanism: inducing ROS formation (Kohanski, Dwyer, Hayete, Lawrence, & Collins, 2007). Thus, we were prompted to examine whether Ino‐1 plays a role in resisting bactericidal antibiotics using relevant *C. glutamicum* strains cultivated in LB broth containing different antibiotics at various concentrations. As shown in Figure 2, all the ROS‐inducing antibiotics tested (ciprofloxacin, streptomycin, vancomycin, and neomycin) displayed significant growth inhibition for the Δ*ino‐1*(pXMJ19) strains, whose inhibitory degree was significantly higher than that for the WT strains. The sensitivity of the Δ*ino‐1*(pXMJ19) strains to these antibiotics has also been reported in MSH‐deficient *C. glutamicum* mutants (Liu et al., 2013). In addition, as a control, no significant difference in growth was observed between WT(pXMJ19) and Δ*ino‐1*(pXMJ19) strains exposed to the ROS production‐unstimulating bacteriostatic antibiotic penicillin. However, more surprising is that the WT(pXMJ19) strain grew significantly better than Δ*ino‐1*(pXMJ19) strains in conditions challenged with rifamycin SV, which also belongs to the ROS production‐unstimulating bacteriostatic antibiotic. Together, these findings demonstrate that Ino‐1 plays an important role in protecting *C. glutamicum* against ROS‐inducing oxidants, alkylating agents, bactericidal antibiotics, and heavy metal stress.

### 3.2 Ino‐1 reduces ROS levels under ROS‐inducing xenobiotic agents stress

**Figure 3:**
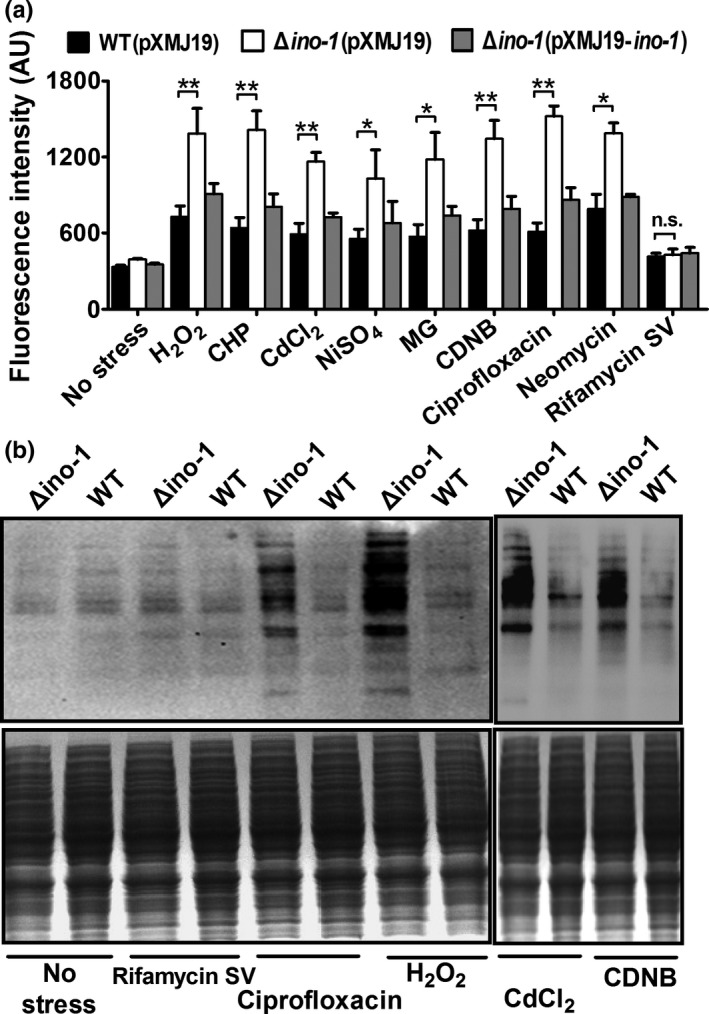
The mutant lacking Ino‐1 increases ROS production under oxidative stress conditions. (a) The intracellular ROS levels in *C. glutamicum* WT (pXMJ19), Δ*ino‐1* (pXMJ19), and Δ*ino‐1*(pXMJ19‐*ino‐1*) strains were measured using the DCF fluorescence assay after exposure to the reagents indicated. Mean values with standard deviations (error bars) from at least three independent experiments are shown. n.s.: not significant. *p ≤ 0.05. **p ≤ 0.01. (b) The mutant lacking Ino‐1 had increased protein carbonyl content under oxidative stresses. The protein carbonyl contents were analyzed using western blotting with the antidinitrophenyl antibody (upper panel). A parallel run was stained with Coomassie Brilliant Blue (bottom panel). The total proteins were extracted from the wild‐type and Δ*ino‐1* mutant cells

Since MSH is a key nonenzymatic antioxidant that protects the cells from ROS damage by directly scavenging free radicals and by serving as a cofactor for antioxidant enzymes, we were prompted to investigate whether Ino‐1 plays a role in removing ROS under stress conditions (Si, Xu, et al., 2015; Si, Zhang, et al., 2015). The ROS levels in vivo were detected using DCFH‐DA, a membrane permeable dye which passively diffuses into cells (Si et al., 2016). After exposure to ROS‐inducing H_2_O_2_, CHP, CdCl_2_, NiSO_4_, MG, CDNB, ciprofloxacin, and neomycin, the *ino‐1* deletion mutant showed obviously higher ROS levels than the WT(pXMJ19) and complement Δ*ino‐1*(pXMJ19‐*ino‐1*) strains (Figure 3a). However, for both the WT(pXMJ19) and Δ*ino‐1*(pXMJ19) strains, there was no difference in the ROS levels between the rifamycin SV‐treated and SV‐untreated strains. In addition, no increase in ROS levels was observed in the Δ*ino‐1*(pXMJ19) strain exposed to rifamycin SV compared with the WT(pXMJ19) strain, which did not correspond to the survival rate observed between the WT(pXMJ19) and Δ*ino‐1*(pXMJ19) strains under rifamycin SV treatment. The result might be because rifamycin SV directly reacted with MSH to form MSH *S*‐conjugates that were then cleaved by amidase to detoxify them independently from ROS production (Kohanski et al., 2007). Together, these results demonstrate that the *ino‐1* gene confers robustness to *C. glutamicum* by being involved in the ROS reduction triggered by multiple stresses.

ROS escaping from the antioxidant defense system more easily reacts with the cysteine thiol groups of proteins, resulting in reversible inter‐ or intraprotein disulfides (PrSSPr, PrSSPr), and mixed disulfides with LMW thiols, and irreversible carbonylation (Nystrom, 2005; Ying, Clavreul, Sethuraman, Adachi, & Cohen, 2007). To test whether Ino‐1 functions in reducing the oxidative damage suffered by proteins under stress conditions, carbonyl groups on total proteins isolated from WT and Δ*ino‐1* strains under adverse stresses were derivatized with 2,4‐dinitrophenyl hydrazine (DNPH) and determined using a western blot with the anti‐DNP antibody. As shown in Figure 3b for both the WT and Δ*ino‐1* strains, rifamycin SV treatment only has background‐level carbonylation, similar to that of the untreated control group. However, exposure to ROS‐producing agents, such as ciprofloxacin, H_2_O_2_, CdCl_2_, and CDNB, led to more carbonylation than that in the untreated control group. In addition, clearer protein carbonyl groups were detected in the Δ*ino‐1* strains after exposure to ROS‐generating agents compared to that in the WT strains (Figure 3b).

### 3.3 Ino‐1 deletion affects growth on glucose under normal conditions to a certain extent

Ino‐1 was involved in the synthesis of the cell wall and MSH biosynthesis. Thus, we were prompted to examine whether the growth of the Δ*ino‐1* mutant was affected on glucose. As shown in Supporting Information Figure S2A, the *ino‐1* gene deletion caused an obvious reduction of the growth of strains in the presence of glucose, in agreement with the phenomenon that inactive Ino‐1‐containing *C. glutamicum* cultured on glucose displays impaired growth compared with the WT (Baumgart et al., 2013). The result indicates that the absence of the *ino‐1* gene is tolerated to some extent, probably due to the presence of paralogous genes in *C. glutamicum*. Our results were obviously different from the result of *M. tuberculosis ino1* (Huang & Hernick, 2015). Growth was negative for *M. tuberculosis ino1* mutants in inositol‐free media, and *M. tuberculosis ino1* mutants failed to be isolated using lower concentrations of inositol on glucose media. In addition, we found that no differences were observed between the WT and Δ*ino‐1* strains in terms of growth when the cells were cultivated with *mIno* instead of glucose as the carbon source, either under stress or under normal conditions (Supporting Information Figure S2B).

### 3.4 Ino‐1 plays an important antioxidant role under oxidative stress conditions

**Figure 4:**
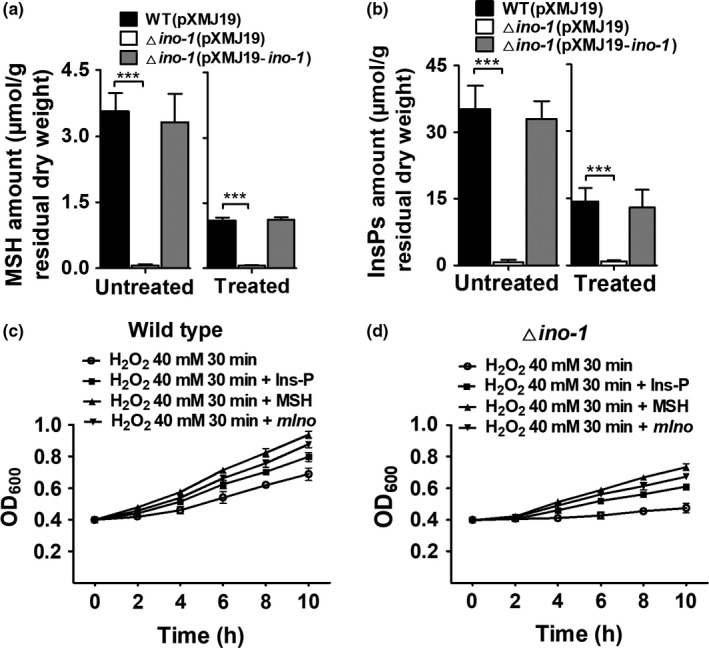
Oxidative stress caused the absence of mycothiol (MSH) and *myo‐inositol‐phosphate* (Ins‐P). (a) Concentration of mycothiol (MSH) in *C. glutamicum*. After the WT (pXMJ19), Δ*ino‐1* (pXMJ19), and Δ*ino‐1* (pXMJ19‐*ino‐1*) strains were exposed to H_2_O_2_ (100 mM) for 30 min, the MSH content was measured as described in the Section 2. Mean values with standard deviations (error bars) from at least three repeats are shown.***p ≤* *0.001. (b) *myo*‐inositol‐phosphate (Ins‐P) content was measured in vivo. After the WT (pXMJ19), Δ*ino‐1* pXMJ19), and Δ*ino‐1* (pXMJ19*‐ino‐1*) strains were grown to 1.6 (OD_600_) in MM media containing glucose, 100 mM H_2_O_2_ was added. After 30 min treatment, the Ins‐P contents were measured using the method described in the Section 2. Mean values with standard deviations (error bars) from at least three repeats are shown.***p ≤* *0.001. (c) Growth phenotypes of the WT and Δ*ino‐1* strains were treated with 40 mM H_2_O_2_ at an OD_600_ of 0.4. The cultures continued to be incubated for 10 hr, and the OD_600_ was measured at 2‐hr intervals. Similar results were obtained in three independent experiments, and the data shown are from one representative experiment conducted in triplicate. (d) Oxidative stress caused *myo*‐inositol‐phosphate (Ins‐p) auxotrophy that was abolished by *myo*‐inositol‐phosphate (Ins‐p) addition. MSH, Ins‐P, and *mIno* were added 30 min after exposure to stress. Similar results were obtained in three independent experiments, and the data shown are from one representative experiment conducted in triplicate

Previous studies have shown that *myo*‐inositol‐phosphate (Ins‐P), synthesized from glucose‐6‐phosphate (Glc‐6P) via Ino‐1 catalysis, was a key precursor substrate of MSH biosynthesis, and MSH can protect *C. glutamicum* cells against oxidative stress (Bachhawat & Mande, 1999; Krings et al., 2006; Newton et al., 2008). Therefore, we inferred that the lack of the *ino‐1* gene caused the absence of Ins‐P and MSH resulting in the oxidative sensitivity phenotype. As expected, the MSH and Ins‐P contents of the Δ*ino‐1*(pXMJ19) strains based on the MSH quantitative standard curve (Supporting Information Figure S3) were near zero under normal conditions, while WT(pXMJ19) strains had a very high content (Figure 4a,b). In addition, the MSH and Ins‐P contents in the Δ*ino‐1*(pXMJ19) strains could almost be fully restored by pXMJ19 plasmid‐based expression of the wild‐type *ino‐1* gene (Figure 4a,b), which was in line with previous findings on inositol and MSH content in a mutant with extremely reduced *ino‐1* expression (Baumgart et al., 2013). More importantly, the MSH and Ins‐P measurements showed an extreme reduction in the WT(pXMJ19) and Δ*ino‐1*(pXMJ19‐*ino1*) strains under oxidative stress compared to those under normal conditions (Figure 4a,b). These results indicated that the Ino‐1 protein plays an important role in the process of antioxidation through cellular MSH levels in *C. glutamicum*.

To further clarify the roles of Ino‐1 under stress conditions, we investigated the duration of the growth lags induced by oxidative stress in the cells growing in glucose‐minimal media in the presence or absence of Ins‐P, MSH, and *mIno*. Forty millimolars of H_2_O_2_ applied could reduce the growth rate of the wild‐type *C. glutamicum* but under sublethal concentrations (Supporting Information Figure S4). One hundred micromolar extracellular Ins‐P, MSH, or *mIno* was added after the cells were treated with 40 mM H_2_O_2_ for 30 min (Figure 4c,d). As shown in Figure 4c,d, the Δ*ino‐1* strain growing in glucose‐minimal media without Ins‐P, MSH, and *mIno* went through a significant inhibition of growth compared with the WT when it was challenged with H_2_O_2._ In addition, the degree of growth inhibition upon being challenged with H_2_O_2_ was significantly higher than that under normal conditions. The findings indicated that Ino‐1 primarily functions in resistance to oxidation in vivo under stress conditions. Notably, 40 mM H_2_O_2_‐treated Δ*ino‐1* strains grown in glucose‐minimal media containing Ins‐P, MSH, and *mIno* significantly resumed growth that was almost equivalent to that of the H_2_O_2_‐untreated wild‐type *C. glutamicum*. These results were further confirmed by the observation that the content of Ins‐P in the H_2_O_2_‐treated cells was lower than that in the untreated cells (Figure 4a,b). The growth experiments suggested that Ins‐P became a key constraining factor under oxidative stress and that the bottleneck to reopen certain metabolic processes was that the strains regained the activity of Ino‐1.

### 3.5 Molecular characterization of protein Ino‐1

Based on a BLAST search and genome sequence analysis, the gene encoding a putative *myo*‐inositol‐1‐phosphate synthase (*ino‐1*) was identified, which was located in the *C. glutamicum* genome (GenBank accession No. NC003450) from 3,197,413 bp to 3,198,504 bp and encoded a protein of 363 amino acid residues with a theoretical molecular mass of 39.2 kDa and a pI of 4.51. The amino acid sequence of Ino‐1 shares 19%, 15%, and 15% identities with the Ino‐1 from *Pseudomonas pickettii* PKO1, *Bacillus thermoglucosidasius* A7, and *Bacillus stearothermophilus* BR219, respectively (Supporting Information Figure S5).

**Figure 5:**
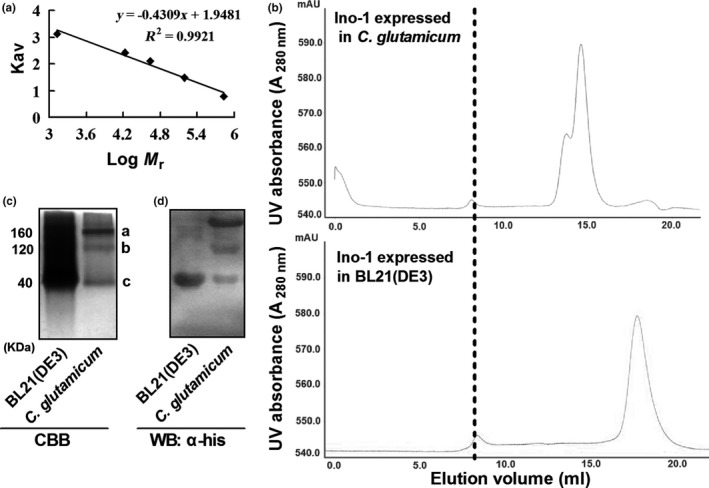
Molecular mass determination of the purified Ino‐1. (a) Molecular weight standard curve. Molecular weight standards from small to large weight: thyroglobulin (bovine) (670 kDa), γ‐globulin (bovine) (158 kDa), ovalbumin (chicken) (44 kDa), myoglobin (horse) (17 kDa), and vitamin B_12_ (1.35 kDa). (b) Gel filtration of Ino‐1. Molecular weight of the purified Ino‐1 from *C. glutamicum* (upper) and BL21(DE3) (lower) was determined using the molecular weight standard curve as described in the Section 2. The dashed line crossing the first peak in both elution profiles represents the Dextran 2000. (c) The purified recombinant Ino‐1 proteins from *C. glutamicum* and BL21(DE3) were mixed with the loading buffer [250 mM Tris–HCl (pH 6.8), 0.5% bromophenol blue (BPB), and 50% (v/v) glycerol], separated on 12% PAGE without SDS buffer (pH 8.8) and stained with Coomassie Brilliant Blue R‐250. M, molecular weight marker. (d) The purified recombinant Ino‐1 proteins from *C. glutamicum* and BL21(DE3) were mixed with the loading buffer [250 vmM Tris‐HCl (pHv6.8), 0.5% bromophenol blue (BPB), and 50% (v/v) glycerol], separated on 12% PAGE without SDS buffer (pH 8.8), and analyzed using western blotting with anti‐his antibody

Previous studies showed that the recombinant expression of *M*.* tuberculosis* proteins in *E. coli* resulted in improperly folded trimerical proteins that had a solution molecular weight of 140 kDa, which was equivalent to ~3.5 monomers. However, recombinant expression of the proteins using an *M. smegmatis* host indicated that they existed as a tetramer–hexamer (Bashiri & Baker, 2015; Goldstone, Moreland, Bashiri, Baker, & Shaun, 2008). Therefore, the solution molecular weight of recombinant Ino‐1 expressed in BL21(DE3) and *C. glutamicum* were determined by gel filtration chromatography, native PAGE, western blotting with anti‐his antibody, and MALDI‐TOF MS‐MS after in‐gel digestion (Figure 5a–d). According to the standard curve (Figure 5a) and the result from gel filtration chromatography experiments (Figure 5b, lower panel), the Ino‐1 purified from BL21(DE3) has a solution molecular weight of 40 kDa, suggesting that Ino‐1 was present as a monomer in solution, consistent with the predicted Mr value deduced from its amino acid sequence. Ino‐1 purified from *C. glutamicum* had three peaks corresponding to a molecular mass of 40, 120, and 160 kDa, respectively, indicating that the Ino‐1 expressed in *C. glutamicum* existed as monomers, trimers, and tetramers (Figure 5b, upper panel). The existing state of the Ino‐1 proteins in BL21(DE3) and *C. glutamicum* was also confirmed by native PAGE, western blotting with anti‐his antibody, and MALDI‐TOF MS‐MS after in‐gel digestion (Figure 5c,d).

### 3.6 Enzymatic characterization of the protein Ino‐1

**Figure 6:**
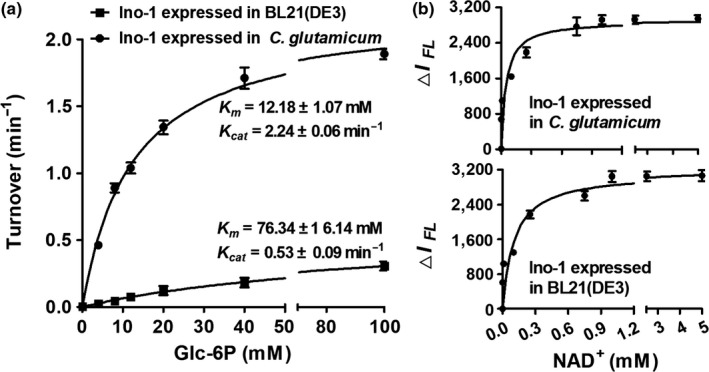
Activity of Ino‐1. (a) Relative activity of Ino‐1 from expression in BL21(DE3) and *C. glutamicum* using the periodate assay. The reaction mixture contained 100 mM Tris‐acetate buffer (pH 7.5), 1 μM Ino‐1, 0–100 mM glucose‐6‐phosphate, 500 μM NAD ^+^, 20 mM NH_4_Cl, and 50 μM ZnSO_4_ at 37°C for 1 hr to gain the Ins‐P. The amount of inositol phosphate formed in the mixture was measured with 0.2 M NaIO_4_ as described in the Section 2. The data were presented as the means of the values obtained from three independent assays. Kinetic parameters were calculated by nonlinear regression using the program GraphPad Prism 5. (b) NAD ^+^ binding to Ino‐1, NAD ^+^ (0–5 mM) binding to Ino‐1 (40 μM) from BL21(DE3) (upper panel) and *C. glutamicum* (lower panel) were determined at pH 7.5 by monitoring the decrease in intrinsic fluorescence (Ex. 280 nm, Em. 334 nm) as described in the Section 2

Unfortunately, the Ino‐1 protein expressed in BL21(DE3) had no activity, consistent with a recent report that a soluble recombinant *M. tuberculosis* Ino‐1 protein yielded in BL21(DE3) had no significant activity with the Glc‐6P substrate (Figure 6a, left panel). To probe whether the lack of activity is attributed to expression in the BL21(DE3) host, we constructed the pXMJ19‐His_6_‐*ino‐1* expression vectors and purified them from the Δ*ino‐1*(pXMJ19) strains. As shown in Figure 6a, the apparent affinity of the Ino‐1 purified from *C. glutamicum* toward Glc‐6P was significantly higher than the value determined with the Ino‐1 purified from BL21(DE3). For example, the *k*_cat_ value of the Ino‐1 purified from *C. glutamicum* and BL21(DE3) toward Glc‐6P was 2.24 ± 0.06 min^−1^ and 0.53 ± 0.08 min^−1^, respectively, while the respective *K*_m_ values were 12.18 ± 1.07 mM and 76.34 ± 1 6.14 mM. These correspond to a catalytic efficiency of 1.8 × 10^5^ and 0.7 × 10^4^ per M per min, respectively. The Ino‐1 purified from *C. glutamicum* showed significant activities with Glc‐6P as substrates using the periodate assay compared to that purified from BL21(DE3), which is higher than the specific activities for Ino‐1 from *M. tuberculosis*.

Since Ino‐1 is known to utilize NAD^+^ as the cofactor, and *M. tuberculosis* Ino‐1 was capable of binding NAD^+^ (Huang & Hernick, 2015), which was highly homologous to *C. glutamicum* Ino‐1 (showing 75% amino acid identities, Supporting Information Figure S5), we probed whether the purified Ino‐1 proteins from *C. glutamicum* and BL21(DE3) were capable of binding NAD^+^ and whether there were differences in the affinity between the Ino‐1 purified from *C. glutamicum* and that from BL21(DE3) by monitoring the decrease in the intrinsic fluorescence of Ino‐1 upon NAD^+^ binding. As shown in Figure 6b, the apparent *K*_*D*_ value for NAD^+^ binding to Ino‐1 from BL21(DE3) (21 ± 3.6 μM) was observed, while it is weaker than that from *C. glutamicum* (4.7 ± 0.8 μM). The results indicated that recombinant expression in BL21(DE3) yielded a soluble protein capable of binding the NAD^+^ cofactor. However, it has no significant activity with the Glc‐6P substrate (Figure 6a). In contrast, the recombinant expression in *C. glutamicum* yielded a functionally active protein when Glc‐6P and NAD^+^ were used as substrate.

## 4 DISCUSSION

Ins‐1P is primarily formed by Ino‐1 catalyzing Glc‐6P, which has many functions, including the promotion of fat metabolism, the formation of the cell membrane, and protein synthesis (Bachhawat & Mande, 1999; Krings et al., 2006). Thus, Ino‐1 plays a key role in the growth and survival of organisms. However, most studies on Ino‐1 have been conducted in eukaryotes, and few have been conducted in prokaryotes (Gumber, Loewus, & Loewus, 1984; Mishra et al., 2015; Nystrom, 2005; Zhai et al., 2015). In this study, we have expanded the list by clearly demonstrating that Ino‐1 plays an important role in oxidative stress resistance in *C. glutamicum*, a well‐known industrial bacterium for the production of amino acids and various organic acids. The mutation of Ino‐1 in *C. glutamicum* made it show significantly increased bacterial sensitivity to oxidants, alkylating agents, heavy metals, and antibiotics, and such sensitivity was nearly restored to wild‐type levels upon complementation with the *ino‐1* gene (Figure 1). In addition, intracellular ROS accumulation and the associated protein carbonylation were significantly higher (compared with the wild‐type) in the Δ*ino‐1* mutant exposed to oxidative stress induced by various agents, suggesting that Ino‐1 plays a vital role in the protection against oxidative stress.

Mycothiol (MSH), the dominant low‐molecular‐weight thiol (LMWT) restricted to the high‐(G+C)‐content gram‐positive *Actinobacteria*, has been regarded as a functional equivalent of glutathione (GSH) in these species and plays important roles in maintaining cytosolic redox homeostasis and in adapting to ROS (Dalle‐Donne, Rossi, Colombo, Giustarini, & Milzani, 2009; Gao, Bedhomme, Veyel, Zaffagnini, & Lemaire, 2009). To date, MSH has been reported to be involved in the detoxification of a broad range of poisonous chemicals, such as oxidants, electrophiles, antibiotics, aromatic compounds, heavy metals, and ethanol (Bachhawat & Mande, 1999; Dalle‐Donne et al., 2009; Gao et al., 2009). In *C. glutamicum*, MSH appears to detoxify endogenously generated antibiotics and reactive intermediates (Krings et al., 2006). In addition, in *C. glutamicum*, Ino‐1 is the primary precursor enzyme during the synthesis of MSH. It catalyzes Glc‐6P for the Ins‐P that is involved in MSH synthesis. Therefore, we speculated that Ino‐1 plays an antioxidative role by affecting cellular MSH levels in *C. glutamicum,* and the supersensitive phenotype of *ino‐1* mutants to adverse stress may result from the reduction in the MSH levels. As expected, our experimental data demonstrated that Ins‐1P auxotrophy was induced by oxidative stress. In addition, regardless of H_2_O_2_ treatment, the lack of the *ino‐1* gene caused the absence of Ins‐P and MSH contents in *C. glutamicum,* consistent with the results of Baumgart et al. (2013). These results indicated that *C. glutamicum* has an antioxidant function with the help of abundant MSH, which is involved in ROS detoxification.

Sequence alignment analysis revealed that the Ino‐1 of *C. glutamicum* shared many conservative amino acids with those of different species. *C. glutamicum* Ino‐1 was highly similar to the *M. tuberculosis* Ino‐1. As expected, the Ino‐1 purified from BL21(DE3) has a solution molecular weight of 40 kDa, consistent with the predicted Mr value deduced from its amino acid sequence. Interestingly, there was a difference in the solution molecular weights between the recombinant Ino‐1 proteins expressed in BL21(DE3) and that in *C. glutamicum*, which was similar with the observation made with *M. tuberculosis* Ino‐1. In addition, recombinant expression in *C. glutamicum* yielded a functionally active protein but not in BL21(DE3). The phenomena might be explained that the variations in protein folding/assembly caused the activity differences when the proteins were expressed in BL21(DE3) and *C. glutamicum*. The results indicated that the activity for the protein expressed in *C. glutamicum* was attributed to folding the enzyme in a catalytically competent conformation.

In summary, this study showed that the *ino‐1* gene in *C. glutamicum* contributes to survival rates in response to environmental oxidative stress. Our insights into the versatile protective roles of Ino‐1 in *C. glutamicum* provide a promising strategy to engineer robust industrial strains in the future.

## CONFLICT OF INTEREST

The authors declare no conflict of interest.

## AUTHOR CONTRIBUTIONS

M.S., C.C., and T.S. conceived the project. M.S., C.C., T.S., K.Q., B.Z., G.Z., and J.F. performed the experiments. M.S. and C.C. analyzed the data. M.S., C.C., and T.S. wrote the paper.

## Data Availability

All data supporting during this study are included in the results section or Supplementary Information.
