# Supplementary material for: Myo‐inositol‐1‐phosphate synthase (Ino‐1) functions as a protection mechanism in Corynebacterium glutamicum under oxidative stress
Source: Microbiologyopen. 2018 Oct 1;8(5):e00721. doi: 10.1002/mbo3.721 (PMC6528642; doi:10.1002/mbo3.721)
Supplement: Supplementary file 1 [file MBO3-8-e00721-s001.doc]

**Supplemental Data**

**Table S1. Bacterial strains, plasmids and Primers used in this study.**

| **Strains or plasmids** | **Relevant genotype description** | **References** |
| --- | --- | --- |
| **Strains** | | |
| ***Corynebacterium glutamicum*** | | |
| RES167 | Restriction-deficient mutant of ATCC13032, Δ(*cglIM-cglIR-cglIIR*) | 1 |
| Δ*ino-1* | *ino-1* deleted in RES167 | This study |
| Δ*ino-1*(pXMJ19-*ino-1*) | Complementation of *ino-1* in Δ*ino-1* | This study |
| Δ*ino-1*(pXMJ19-His_6_-*ino-1*) | Expression of *ino-1* in Δ*ino-1* | This study |
| BL21(DE3)( pET28a-*mdmpi*) | Expression of *mdmpi* in BL21(DE3) | 2 |
| BL21(DE3)( pET28a-*fph*) | Expression of *fph* in BL21(DE3) | 2 |
| BL21(DE3)( pET28a- *g12d*) | Expression of *g12d* in BL21(DE3) | 2 |
| ***E. coli*** | | |
| BL21(DE3) | *E. coli* expression host, *hsdS gal* (*λc*I*ts*857 *ind-l* *Sam7 nin-*5 *lac UV5-*T7 gene 1) | Novagen |
| JM109 | *recA1 supE44 endA1 hsdR17 gyrA96 relA1 thi* Δ(*lac-proAB*)F′(*traD36 proABlacI*^q^ *lacΔZM15*) | Stratagene |
| **Plasmids** | | |
| pK18*mobsacB* | Suicide plasmid carrying *sacB* for selecting double crossover in *C. glutamicum*, Km^r^ | 3 |
| pK18*mobsacB-*Δ*ino-1* | Construct used for in-frame deletion of *ino-1* | This study |
| pXMJ19 | Shuttle vector (*P_tac_ lacI^q^ pBL1 oriV_C. glutamicum_* pK18 *oriV_E. coli_*) | 4 |
| pXMJ19-*ino-1* | *ino-1* cloned into pXMJ19 for complementation | This study |
| pXMJ19-His_6_ | Modified pXMJ19 containing N-terminal hexahistidine tag | 5 |
| pXMJ19-His_6_-*ino-1* | *ino-1*cloned into pXMJ19-His_6_ for overexpression | This study |
| pET28a | Expression vector with N-terminal hexahistidine affinity tag | Novagen |
| pET28a*-ino-1* | *ino-1* in pET28a | This study |
| **Primiers** | **5’-3’ sequence** |  |
| Ino-1-F | CCCAAGCTTATGAGCACGTCCACCATCAGGGTTG(Hind III) | For cloning *ino-1* wild type and variants into pXMJ19, pXMJ19-His_6_ and pET28a |
| Ino-1-R | TGCTCTAGATTACGCCTCGATGATGAATGCCTCT(Xbal I) |
| DIno-1-F1 | TGCTCTAGAGACCAATGACATCGTTGAGGTTGCT(Xbal I) | To generate pK18*mobsacB-*Δ*ino-1* |
| DIno-1-R1 | CAACCCTGATGGTGGACGTGCTCAT |
| DIno-1-F2 | ATGAGCACGTCCACCATCAGGGTTGACGATGTTGCTCGTGAACGCCTAGA |
| DIno-1*-*R2 | CCGGAATTCTTGATCGCAGCGTGGATCTCCTCTC(EcoR I) |

Underlined sites indicate restriction enzyme cutting sites added for cloning. Letters in italic denote the mutation sites in overlap PCR for site-directed mutagenesis.

1. Tauch, A., Kirchner,O., Loffler, B., Gotker, S., Pühler, A., & Kalinowski, J. (2002) Efficient electrotransformation of *Corynebacterium diphtheriae* with a mini-replicon derived from the plasmid pGA1. Curr. Microbiol. 45, 362–367.
2. Liu, Y., Chen, C., Chaudhry, M.T., Si, M., Zhang, L., Wang, Y., & Shen, X. (2014) Enhancing *Corynebacterium glutamicum* robustness by overexpressing a gene, mshA, for mycothiol glycosyltransferase. Biotechnol Lett. 36(7), 1453-1459.
3. Schäfer, A., Tauch, A., Jager, W., Kalinowshi, J., Thierbach, G. and Pühler, A. (1994) Small mobilizable multi-purpose cloning vectors derived from the *Escherichia coli* plasmids pK18 and pK19: selection of defined deletions in the chromosome of *Corynebacterium glutamicum*. Gene. 145, 69–73.
4. Jakoby, M., Ngouoto-Nkili, C.E., & Burkovski, A. (1999) Construction and application of new *Corynebacterium glutamicum* vectors. Biotechnol. Techniques. 13, 437–441.
5. Si, M., Wang, T., Pan, J., Lin, J., Chen, C., Wei, Y., Lu, Z., Wei, G., & Shen, X. (2016). Overexpression of mycothiol disulfide reductase enhances *Corynebacterium glutamicum* robustness by modulating cellular redox homeostasis and antioxidant proteins under oxidative stress. *Sci. Rep.* 6, 29491.


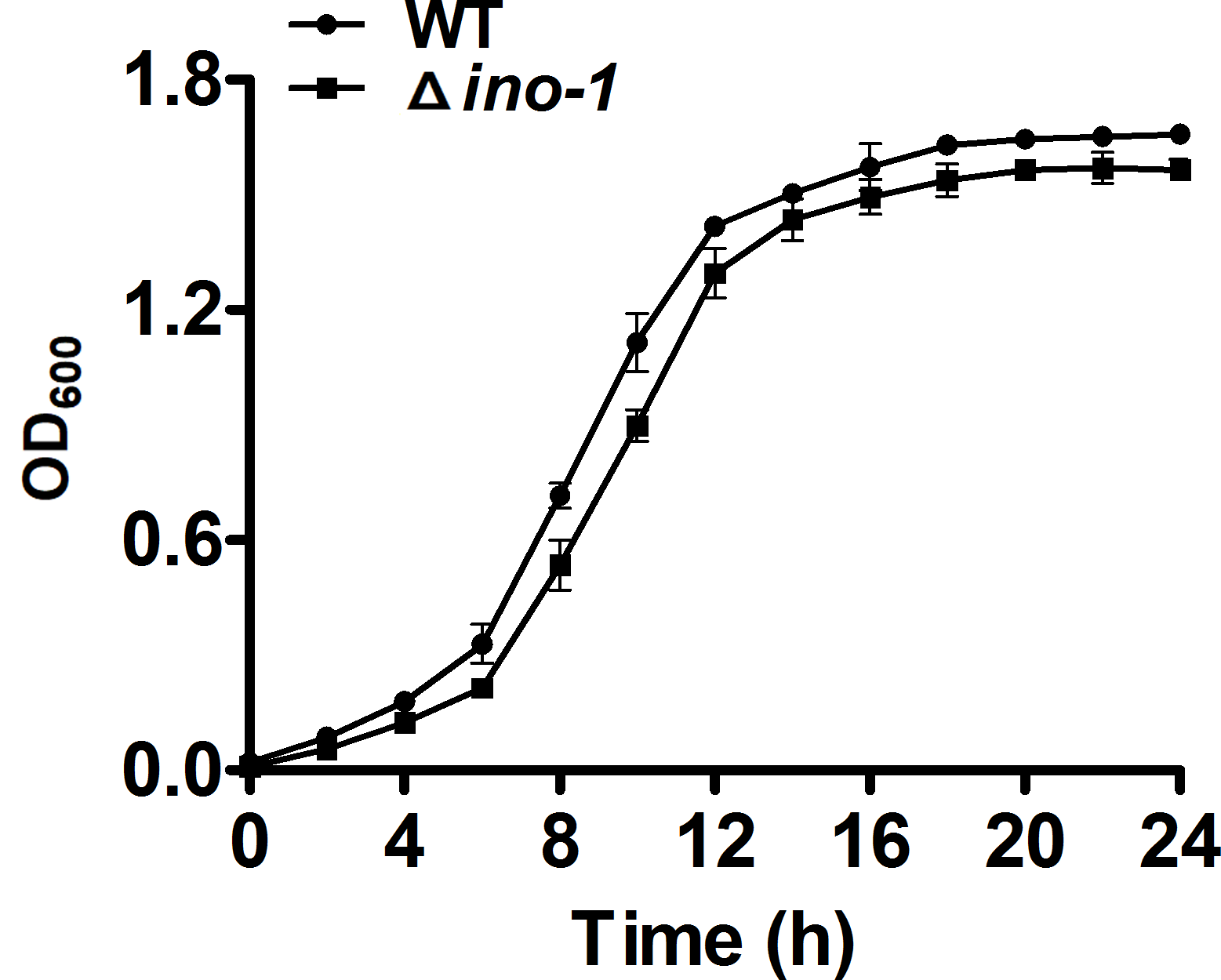


**Figure S1 Deletion of the *ino-1* gene did not affect bacterial growth under normal conditions.** Overnight-grown cultures of *C. glutamicum* (LB broth, 30 °C) were diluted 100-fold with LB medium and the growth in LB was monitored by measuring OD_600_ at indicated time points. Data shown were the average of three independent experiments; error bars indicate SD from three independent experiments.


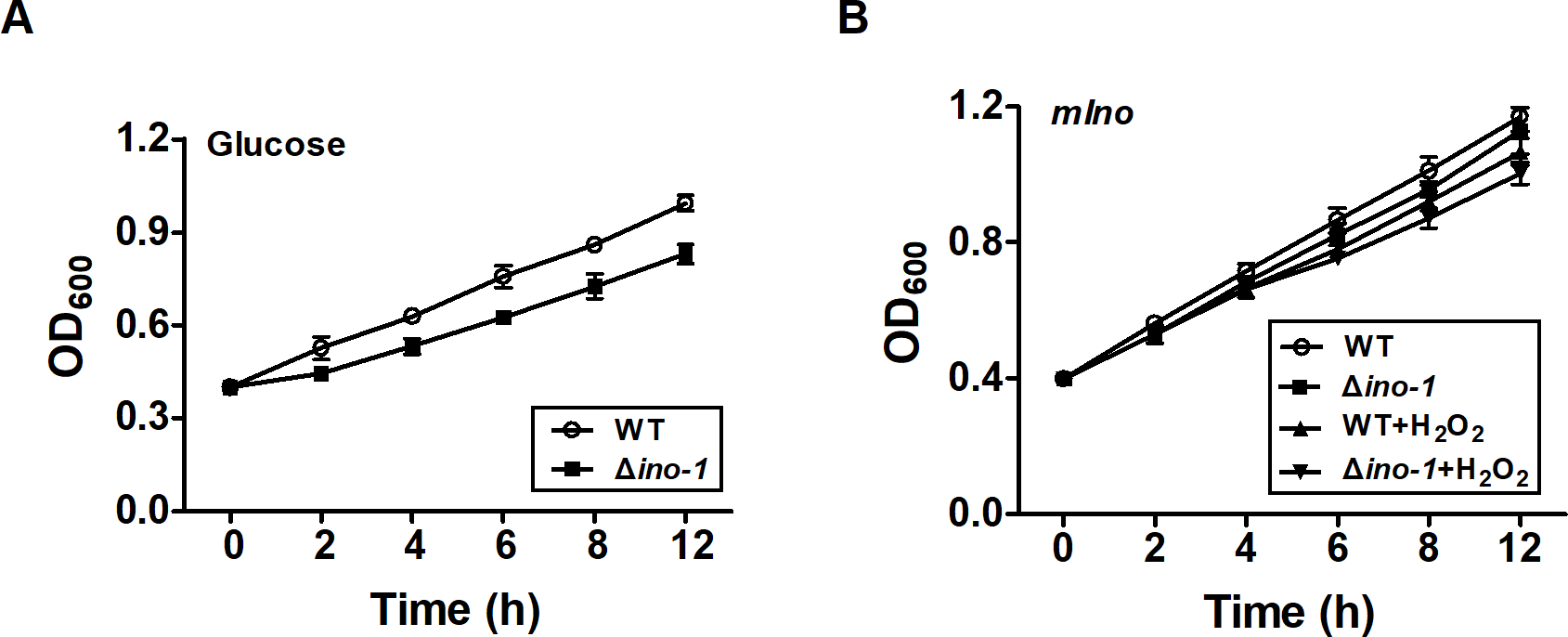


**Figure S2 Growth curves and phenotype of *C. glutamicum* wild-type andΔ*ino-1*.** Results in show the growth from sequential cultures in minimal medium with glucose (A) or *myo-inositol* with or without H_2_O_2_ treatment (B) as carbon source. After reaching an OD_600_ of 1.6, the cells were diluted into fresh medium at an initial OD _600_ of 0.4. Data shown were the average of three independent experiments; error bars indicate SD from three independent experiments.

**
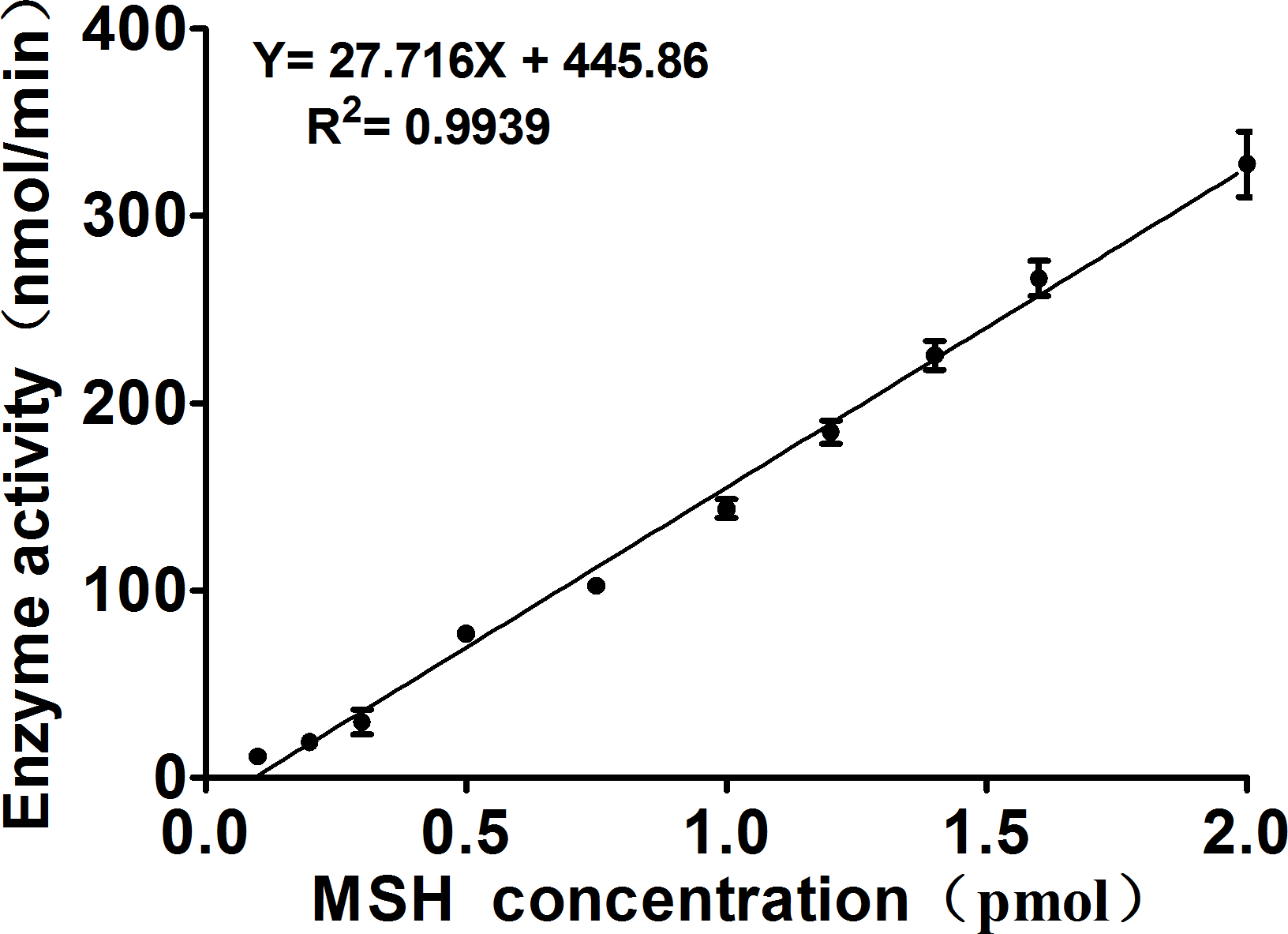
**

**Figure S3 The MDMPI activities were linear to the MSH amounts.**

MSH purified from *C. glutamicum* was used. Data were averages from three parallel measurements. The MSH amount of each spot is 0.1, 0.21, 0.31, 0.49, 0.75, 1, 1.22, 1.43, 1.68, and 2 pmol, respectively.


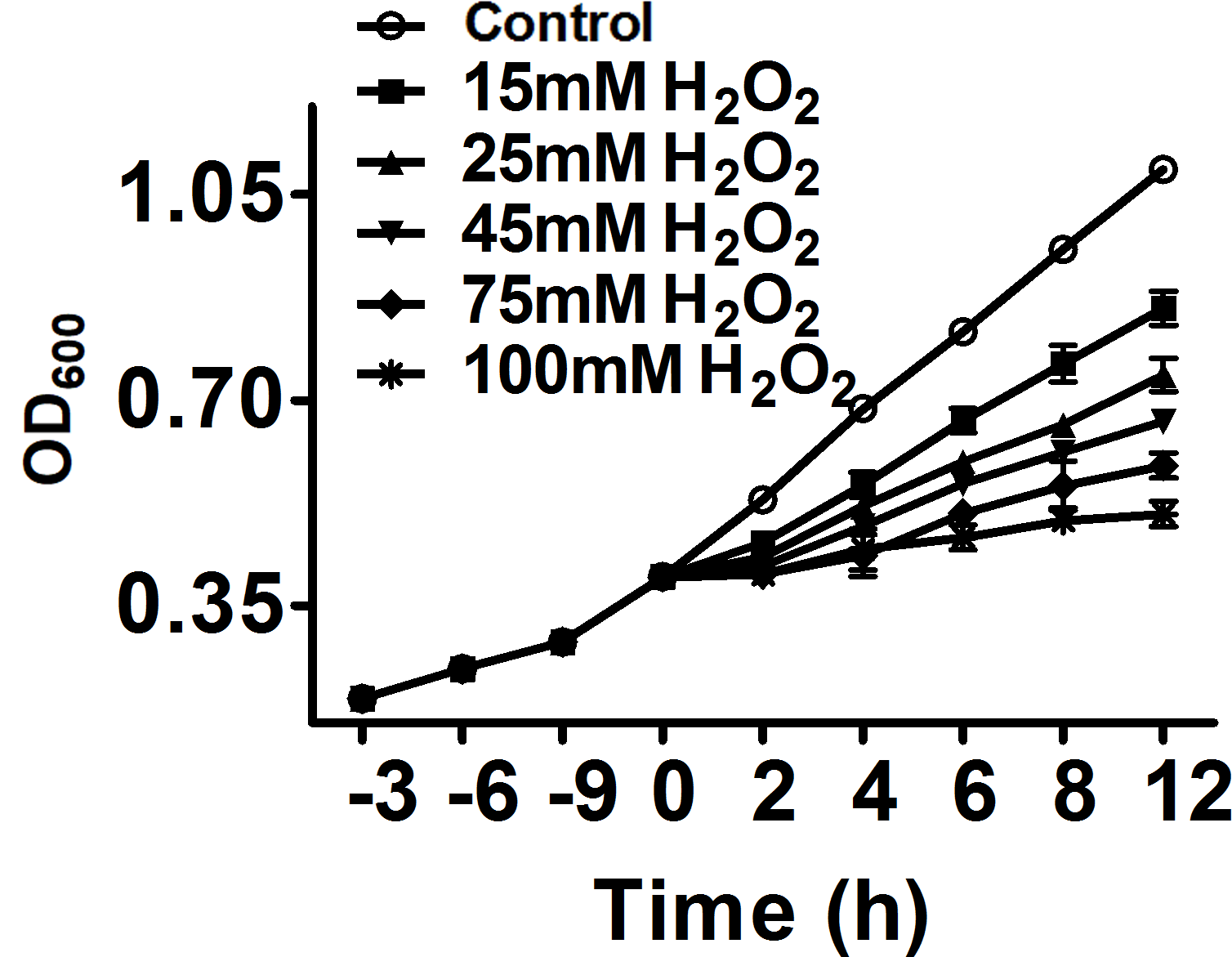


**Figure S4 Growth curves of *C. glutamicum* in response to sub-lethal concentrations of toxins.** Overnight-grown cultures of *C. glutamicum* (LB broth, 30 °C) were diluted 100-fold with LB medium and Cultures were allowed to grow to an OD_600_ of approximately 0.4 and exposed to various concentrations of H_2_O_2_. The cultures continued to be incubated for 10 h, and the OD_600_ was measured in 2 h intervals.


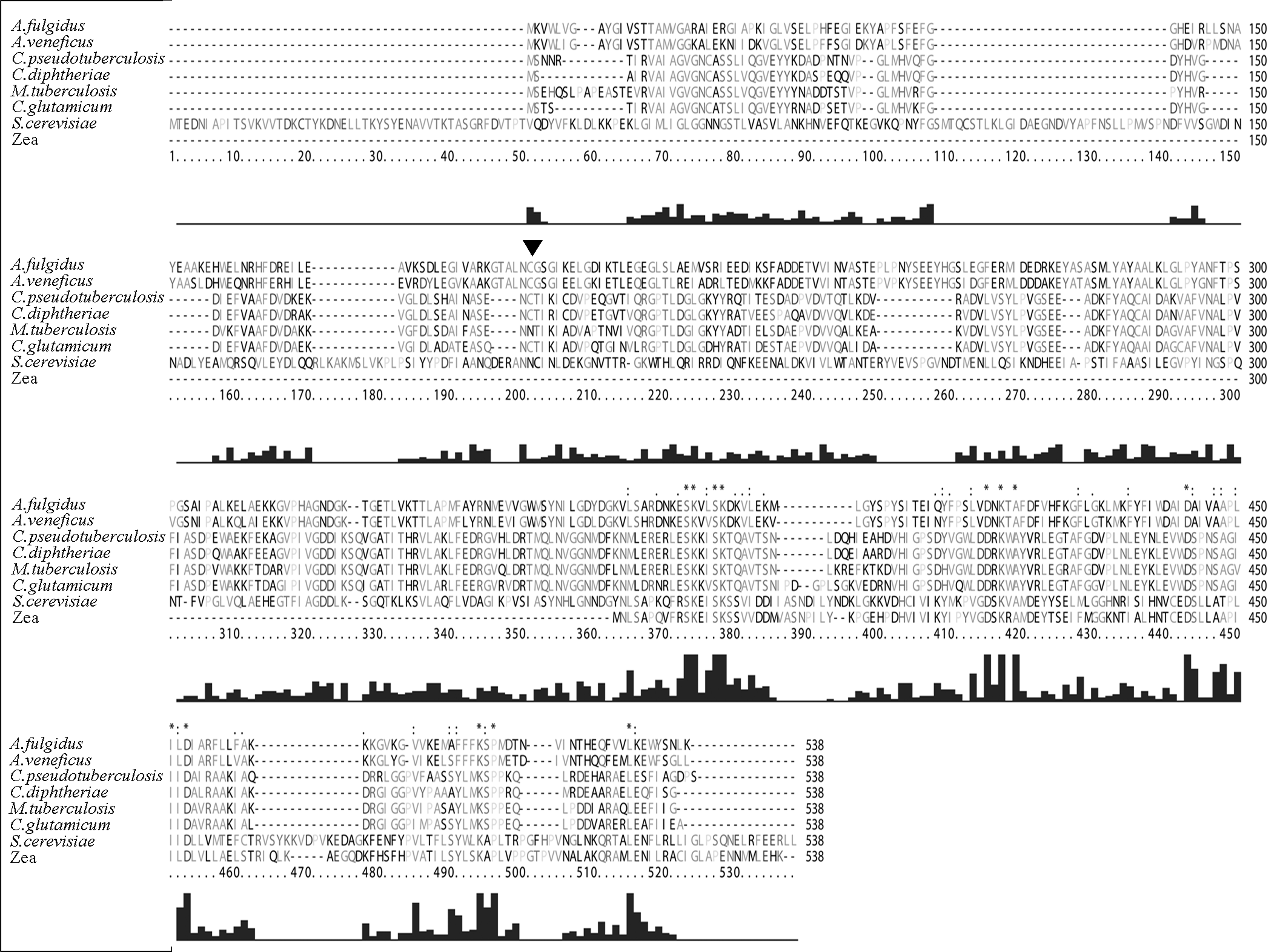


**Figure S5 Multiple sequence alignment of *C. glutamicum* Ino-1 with other representative Ino-1 proteins.**

The locations of the cysteine residues were indicated by filled triangle. Accession numbers: Ino-1 from *Archaeoglobus fulgidus* (O28480), *Archaeoglobus veneficus* SNP6 LOCUS (YP_004342749), *Corynebacterium pseudotuberculosis 267* (AFH50984.1), *Corynebacterium diphtheriae* (KG59180.1), *Mycobacterium tuberculosis* (P9WKI1), *Corynebacterium glutamicum* (CAF18936.1), *Saccharomyces cerevisiae* (P11986), and *Zea* (ACN2623).
